# “I Was Just Like a Sponge, Absorbing All the Wrong Information”: Examining the Role of Social Media in Athletes' Eating Disorder and Recovery Experiences

**DOI:** 10.1002/eat.70088

**Published:** 2026-03-16

**Authors:** Olivia Feng, Lindsay R. Duncan

**Affiliations:** ^1^ Department of Kinesiology & Physical Education McGill University Quebec Canada

**Keywords:** athlete, eating disorder, media literacy, recovery, social media, treatment

## Abstract

**Objective:**

Within sport environments, athletes are exposed to norms that circulate narrow meanings about the body and food, contributing to the high prevalence of eating disorders (EDs). While social media can support ED recovery, it can also further constrain food‐ and body‐related messages for athletes. The purpose of this study was to examine the role of social media in athletes' ED and recovery experiences.

**Method:**

Data were drawn from a broader study on athletes' support networks during ED recovery, where 29 participants (17 athletes, 12 social agents) completed one‐on‐one semi‐structured interviews. For the present study, all discussion of social media was extracted from the interviews and analyzed using reflexive thematic analysis.

**Results:**

We identified four themes. The first theme, *Positive Aspects of Social Media Undermined by Harmful ED Content*, describes how, despite facilitating connection, social media often exacerbates existing food‐ and body‐related pressures for athletes. The second theme, *Establishing a New Relationship With Social Media to Protect ED Recovery*, showcases how athletes applied social media literacy skills. The third theme, *Using Social Media to Challenge ED Stigma and Diet Culture*, describes how some athletes initiated conversations about EDs and countered harmful diet culture messages through their platforms. The fourth theme, *Online Visibility as a Recovery Stressor*, depicts how increasing pressures for athletes to curate a personal brand on social media may reexpose them to ED‐related content.

**Discussion:**

Findings highlight the need for athlete‐specific social media literacy within ED prevention and treatment programs, and for sport organizations and media platforms to support safer online environments.

## Summary

- This study examined the role of social media in athletes' eating disorder and recovery experiences.
- Participants described that social media often exacerbates food‐ and body image‐related pressures athletes face, which can hinder eating disorder recovery.
- Increasing pressure for athletes to cultivate a personal brand on social media can also complicate recovery.
- Our findings highlight the need for athlete‐specific social media literacy interventions and systemic efforts to support safer online environments.

## 1 Introduction

Within sport environments, athletes are exposed to messages and practices that circulate narrow meanings about the body and food, where certain bodies (e.g., lean, toned) are legitimized (Papathomas 2018). These norms contribute to the high prevalence of eating disorders (EDs) among athletes (Papathomas 2018), which are estimated to impact up to 45% of women and 33% of men athletes (Bratland‐Sanda and Sundgot‐Borgen 2013; Karrer et al. 2020). Although appropriate support can facilitate ED recovery (Arthur‐Cameselle and Quatromoni 2014), athletes must navigate additional sociocultural pressures beyond sport.

Social media circulates pro‐ED, diet culture, and fatphobic content linked to negative body image and ED behaviors (Dane and Bhatia 2023). Even ED recovery‐oriented spaces may include triggering content (e.g., specific weights or calories), body shaming comments, and appearance‐focused discourses that hinder recovery (Au and Cosh 2022; Goh et al. 2022). Navigating social media during ED recovery may therefore be particularly complex, as societal messages about health (e.g., “eat less, move more”) can conflict with recovery‐oriented prescriptions (Nikolova and LaMarre 2023). For athletes, social media is a near‐inevitable extension of sport culture, as athletes can be algorithmically exposed to sport‐specific content based on their online engagement (Kapsch 2022) and are increasingly expected to curate their social media profiles to promote personal brands for sponsorships, endorsement deals, and professional opportunities post‐retirement (Doyle et al. 2022). Consequently, athletes face unique pressures when navigating social media that can increase vulnerability to EDs and complicate recovery.

Despite the harms of social media, research suggests it can support ED recovery by fostering community, challenging diet culture, and providing informational and emotional support (Au and Cosh 2022; Goh et al. 2022; Herrick et al. 2021; Hallward et al. 2025). For athletes, different platforms (e.g., Instagram, TikTok, Strava) can also shape experiences in distinct ways. To date, limited research has explored how athletes in ED recovery navigate social media. Therefore, this study examined the role of social media in athletes' ED and recovery experiences.

## 2 Method

### 2.1 Data Collection

This study received ethical approval from McGill University Research Ethics Board (REB23‐11‐005) and is part of a broader study exploring key people within athletes' networks during ED recovery and sport reintegration. We recruited 17 athletes and 12 social agents (i.e., individuals who supported athletes in ED recovery) via purposeful sampling between March 2024 and April 2025 for a semi‐structured interview. Athletes were eligible if they were training for competition or had retired within 3 years after returning to sport following an ED or disordered eating. Social agents were eligible if they held roles identified within athletes' networks (e.g., coach, dietitian) and had supported (professionally or personally) at least one athlete in recovery. Some social agents had relationships with athletes in the study; however, we did not systematically recruit athlete–social agent pairs, interviews were conducted independently, and we do not report identifying connections. Participants' demographic information is provided in Tables S1 and S2.

Interviews explored ED and sporting experiences, and who and what actions can support athletes in ED recovery and reintegration into sports. Social media was not a primary focus but was a topic of substantive discussion in 11 interviews. When mentioned, interviewers probed to understand its perceived role.

### 2.2 Data Analysis

From the larger dataset, we extracted and analyzed all discussion of social media using an inductive approach to reflexive thematic analysis (Braun and Clarke 2021). Athlete and social agent accounts were analyzed as independent perspectives. O.F. first familiarized herself with athletes' data, then identified common patterns within these data. This process was repeated with social agents' data, and initial codes were compared across groups to remain sensitive to differences in experiences (e.g., athletes' lived experiences vs. social agents' observations). While many codes overlapped (e.g., harms of social media), only athletes described branding pressures. Initial codes were discussed with LRD to refine interpretations. Through an iterative process, initial codes were collated to form larger themes, which were refined, defined, and named through ongoing discussion.

Guided by a constructionist paradigm (Guba and Lincoln 1994), we acknowledge that reality is subjective and knowledge is co‐constructed. Both authors have personal histories of ED or disordered eating within sport and are active social media users. We engaged in ongoing reflexive dialogue to critically examine how our positions shaped interpretation.

### 2.3 Involvement of Individuals With Lived Experience

Persons with lived experience were not involved in the study design or execution, or in the preparation of this manuscript.

## 3 Results

We identified four themes that capture the role of social media in athletes' ED and recovery experiences. Pseudonyms are used to protect participants' identities.

### 3.1 Positive Aspects of Social Media Undermined by Harmful ED Content

Many athletes noted that social media can facilitate intentional connection with other athletes and professionals. For example, Caroline (athlete) described receiving emotional and informational support, explaining that she “made a lot of connections… with professionals and people who had been through REDS [relative energy deficiency in sport] and gotten out of it.” Despite these benefits, athletes emphasized that social media simultaneously increases exposure to harmful content that can contribute to EDs and exacerbates sport‐specific pressures that athletes were already subject to in sport, particularly around body ideals, food, and training. Caroline shared: “[Social media] is definitely a source of comparison… it just wasn't always good because I would see the amount [of training] people were doing. And if they can eat like that and do that much, then why can't I?” Peyton (athlete) shared a similar experience:

My eating disorder is really bound up in comparison… that's kind of where it stemmed from… I think that's so common in sport, like you do so much comparison cause you're like, “I wanna be the best”… I know people get [comparisons] from social media, and it's a huge thing in sport…

Social agents also expressed concern about athletes' exposure to unqualified spokespeople or “influencers,” where “the advice [athletes] are getting on social media from absolutely random people is really impactful” (Megan, physiotherapist). Margaret (dietitian) discussed how diet culture content can negatively impact athletes' relationships with food:

Everyone is looking up what other professional athletes are eating online… and those athletes online have a humongous influence, especially if they're posting “oh, I've never eaten pizza.” That resonates. And then an athlete who's like 12, 13, 14, is like, “oh, they're this good and they don't ever eat pizza. I better stop eating pizza.”

Caroline (athlete) emphasized that such messaging is “definitely not healthy, especially in recovery.” Similarly, Carley (athlete) described avoiding content such as “what I eat in a day” videos because “I think subconsciously it does [affect me] a little bit.” These examples illustrate how social media, while offering connection and support, is saturated with messages that can exacerbate existing food‐ and body‐related pressures for athletes, particularly during recovery.

### 3.2 Establishing a New Relationship With Social Media to Protect ED Recovery

To protect recovery, athletes described setting boundaries with social media. Sadie (athlete) explained that platforms such as Strava reinforced unhelpful behaviors during her recovery: “I would've deleted Strava earlier… because I just don't think it was helpful. I think it was feeding into me doing too much and changing my training habits.” Caroline (athlete) emphasized becoming more intentional with who she follows and developing social media literacy skills:

Now I'm a lot more sensible with [social media]… I can read something and be less susceptible to it. I think before I was looking for information to validate the [ED] beliefs… I was just like a sponge, absorbing all the wrong information.

Reese (teammate/friend) echoed this, noting: “The take home message is don't believe everything you see [online].”

Participants noted that social media literacy for athletes should involve recognizing athletes' specific energy and nutritional needs. Carley (athlete) explained being able to now critique the lack of nuance in online discourse:

Social media is definitely a big problem with [EDs] because I know what the general population eats in a day is not what an athlete should be eating… it's all context dependent… social media likes to make very black and white rules.

### 3.3 Using Social Media to Challenge ED Stigma and Diet Culture

Some athletes used social media to challenge ED stigma and counter diet culture messages. Reese, a key supporter in her teammate/friend's ED recovery, spoke about the Instagram account they created together to do so:

I see so much rubbish out there of people saying, “this is what you should do in sport.” Actually, no—you can do whatever… I feel like [our content] is the kind of stuff people want to see. They want to see people being able to improve in sport and be happy in sport with a normal diet. They don't want to see that the only way to get faster, fitter, and stronger is by eating less than 2000 cal a day.

Maya (athlete) described how sharing her recovery on social media prompted supportive responses from other athletes: “I think it fostered a more open environment both within my team and within the international cohort… and hopefully it's reaching beyond that.”

Margaret (dietitian) noted that while “win at all costs” narratives persist in sport culture, athlete advocates are helping to shift these norms:

There's been, and that's maybe the positive side of social media, athletes that have said “people look at me as an endurance athlete and they think I don't have a menstrual cycle, but I do. And I have to have a menstrual cycle otherwise my bones will break.” And so using advocates like that, that are really open and saying… “I'm not this size because I'm restricting.”

### 3.4 Online Visibility as a Recovery Stressor

Athletes specifically described increasing pressure to cultivate a personal brand online, which some perceived as distorting the criteria for athletic success beyond performance and reinforcing comparison‐based stressors linked to ED risk. Katie (athlete) shared her perspectives on this unique pressure:

Athletes are sort of in this bubble of “you are this person, and this is your brand” … like someone can be quite shit and have a million followers on Instagram, and they probably feel the same, if not more, stress and pressure than someone who has a gold medal but has 1000 followers.

For athletes who shared their ED experiences online, social media advocacy introduced emotional demands that at times complicated recovery. Maya (athlete) described feeling overwhelmed when others sought support or disclosed their own ED struggles:

While I'm always very humbled and flattered when that happens… I'm not a clinical psychologist. I can only speak to my own experience… So, I sometimes feel stressed, especially earlier on when [recovery] was all still very new and painful for me.

These examples show how online visibility may place athletes in emotionally taxing roles that risk re‐exposure to ED‐related content and undermine recovery boundaries.

## 4 Discussion

This study examined the role of social media in athletes' ED and recovery experiences. Participants described that social media sometimes fostered openness, potentially supporting shifts in sport culture, which is consistent with research demonstrating that recovery‐oriented social media spaces can reduce ED stigma (Au and Cosh 2022). However, participants also described how ED‐related content on social media often exacerbated existing food‐ and body‐related pressures for athletes, particularly during recovery. This shows that exposure to harmful sport and diet culture messages can influence ED behaviors and hinder recovery, suggesting that navigating social media as an athlete presents unique challenges that may require specific considerations to protect recovery.

Participants discussed social media literacy as central to recovery. While social media literacy has become a target in ED prevention programs (Rodgers et al. 2024), athlete‐specific prevention programs should target such skills as well (Reel et al. 2021; Ricker et al. 2025). Recent programs such as the Young Athlete Body Project (Sundgot‐Borgen et al. 2024) and BodySense (Sport Integrity Canada n.d.) incorporate elements to improve athletes' social media literacy, including identifying photo manipulation techniques and critically evaluating information sources and appearance ideals. However, limited research has examined how addressing social media within ED treatment may support recovery (Rodgers et al. 2024). Our findings also suggest that athlete‐specific social media literacy interventions must address considerations related to active (e.g., self‐branding), as opposed to passive social media engagement, and how this impacts athletes' self‐representation and self‐image.

## 5 Strengths, Limitations, and Future Directions

This study included athletes from diverse sports and social agents in varied roles, enabling nuanced insight into the impact of social media on athletes' sporting and ED experiences. However, because social agents represented varied professional and relational roles, their perspectives reflected differing forms of proximity and expertise. Future research could examine how individuals in specific roles (e.g., clinicians vs. coaches) interpret social media influences differently. Additionally, athletes were limited to adults (18+), and our sample comprised predominantly White/Caucasian women. Future research should examine how social media can be safely leveraged in ED recovery across genders, cultural contexts, sport types, and media platforms particularly relevant to athletes (e.g., Strava). Given athletes described online visibility as a recovery stressor, beyond improving individual athletes' social media literacy, systemic efforts should target sport organizations' policies to promote responsible posting on social media. Social media companies should also continue regulating ED‐related content (e.g., TikTok 2025). Together, these findings underscore the complex and evolving role of social media in athletes' ED recovery, highlighting the need for coordinated individual and systemic strategies to support safer engagement with social media during ED recovery.

## Author Contributions

**Olivia Feng:** conceptualization, investigation, formal analysis, writing – original draft, and funding acquisition. **Lindsay R. Duncan:** conceptualization, formal analysis, writing – review and editing, funding acquisition, and supervision.

## Funding

This work was supported by the Sport Dispute Resolution Centre of Canada’s Abuse‐Free Sport Research Grant Progam, O.F.'s SSHRC Canadian Graduate Scholarship–Doctoral, and O.F.'s McGill Sport Science Doctoral Fellowship.

## Conflicts of Interest

The authors declare no conflicts of interest.

## Supporting information


**Table S1:** Athletes' (*n* = 17) demographic information.

**Table S2:** Social agents' (*n* = 12) demographic information.

## Data Availability

The data that support the findings of this study are available from the corresponding author upon reasonable request.
