# Supplementary material for: “I Was Just Like a Sponge, Absorbing All the Wrong Information”: Examining the Role of Social Media in Athletes' Eating Disorder and Recovery Experiences
Source: Int J Eat Disord. 2026 Mar 16;59(7):1630–4. doi: 10.1002/eat.70088 (PMC13326760; doi:10.1002/eat.70088)
Supplement: Supplementary file 1 — Table S1: Athletes' (n = 17) demographic information. [file EAT-59-1630-s001.docx]

**Supplementary Table 1.** Athletes’ (n = 17) demographic information.^a^

| **Age range** | 18-41 years (M = 25.2) |
| --- | --- |
| **Genders^b^ represented** | 15 women/female  2 men/male |
| **Self-described ethnicities represented** | 15 White/Caucasian  1 Asian  1 Mixed |
| **Sport types represented** | Endurance sports (e.g., distance running, cycling)  Ball sports (e.g., tennis, soccer, basketball)  Aesthetic sports (e.g., gymnastics, figure skating)  Weight-class sports (e.g., boxing, rowing) |
| **Competition levels represented** | Club/local  Varsity  National  International |
| **Eating disorder diagnoses or experiences represented** | Anorexia nervosa  Bulimia nervosa  Binge eating disorder  Avoidant restrictive food intake disorder  Eating disorder not otherwise specified^c^  Disordered eating |

^a^Demographic information was collected from athletes via an online pre-screening questionnaire administered by the lead author.

^b^Athletes self-reported their gender using an open textbox (to avoid forcing participants to identify using pre-determined categories). Some participants used identifiers more typically associated with sex (e.g., male/female) than gender (e.g., man/woman).

^c^Eating disorder not otherwise specified is the category in the Diagnostic and Statistical Manual for Mental Disorders (4^th^ edition; DSM-IV) for eating disorders of clinical severity that do not meet the diagnostic criteria for either one of the two eating disorders (i.e., anorexia nervosa and bulimia nervosa) recognized in the DSM-IV.
