# Supplementary material for: “I Was Just Like a Sponge, Absorbing All the Wrong Information”: Examining the Role of Social Media in Athletes' Eating Disorder and Recovery Experiences
Source: Int J Eat Disord. 2026 Mar 16;59(7):1630–4. doi: 10.1002/eat.70088 (PMC13326760; doi:10.1002/eat.70088)
Supplement: Supplementary file 2 — Table S2: Social agents' (n = 12) demographic information. [file EAT-59-1630-s002.docx]

**Supplementary Table 2.** Social agents’ (n = 12) demographic information.^a^

| **Age range** | 24-64 years (M = 43.2) |
| --- | --- |
| **Genders^b^ represented** | 9 women/female  2 men/male  1 non-binary |
| **Self-described ethnicities represented^c^** | 10 White/Caucasian  1 Mixed |
| **Roles represented** | Partner  Coach  Dietitian  Psychologist  Parent  Friend  Teammate  Physiotherapist |
| **Athletes’ sport type** | Endurance sports (e.g., distance running, cycling)  Ball sports (e.g., rugby)  Aesthetic sports (e.g., gymnastics, artistic swimming)  Weight-class sports (e.g., boxing) |
| **Athletes’ eating disorder diagnoses or experiences** | Anorexia nervosa  Disordered eating |

^a^Demographic information was collected from social agents via an online pre-screening questionnaire administered by the lead author.

^b^Social agents self-reported their gender using an open textbox (to avoid forcing participants to identify using pre-determined categories). Some participants used identifiers more typically associated with sex (e.g., male/female) than gender (e.g., man/woman/nonbinary).

^c^One participant did not provide their ethnicity.
